# Nucleotide excision repair pathway gene polymorphisms are linked to breast cancer risk in a Chinese population

**DOI:** 10.18632/oncotarget.12744

**Published:** 2016-10-19

**Authors:** Bang-shun He, Tao Xu, Yu-qin Pan, Han-jin Wang, William C. Cho, Kang Lin, Hui-ling Sun, Tian-yi Gao, Shu-kui Wang

**Affiliations:** ^1^ General Clinical Research Center, Nanjing First Hospital, Nanjing Medical University, Nanjing, China; ^2^ Department of General Surgery, Nanjing First Hospital, Nanjing Medical University, Nanjing, China; ^3^ Department of Clinical Oncology, Queen Elizabeth Hospital, Hong Kong, China; ^4^ Department of Laboratory Medicine, Nanjing First Hospital, Nanjing Medical University, Nanjing, China

**Keywords:** association study, breast cancer, Chinese females, nucleotide excision repair (NER) pathway, polymorphism

## Abstract

Polymorphisms in nucleotide excision repair (NER) pathway genes are associated with the risk of breast cancer, but the relevance of these associations appeared to vary according to the ethnicity of the subjects. To systemically evaluate the potential associations between NER polymorphisms and breast cancer risk in a Chinese population, we carried out a case-control study on 450 breast cancer patients and 430 healthy controls. Sequenom MassARRAY was used for genotyping, and immunohistochemistry was performed to detect estrogen receptor (ER), progesterone receptor (PR), and human epidermal growth factor receptor 2 (HER-2) expression in tumor tissue. Our results showed that *ERCC1* rs11615 (additive model: OR_adjusted_: 1.36, 95% CI: 1.08-1.71, *p* = 0.009), *XPC* rs2228000 (additive model: OR_adjusted_: 1.39, 95% CI: 1.13-1.72, *p* = 0.002) and *ERCC2/XPD* rs50872 (additive model: OR_adjusted_: 1.32, 95% CI: 1.04-1.67, *p* = 0.021) were associated with an increased risk of breast cancer. Stratified analysis revealed three polymorphisms (rs11615, rs1800975, and rs50872) to be associated with breast cancer in menopausal females. Three polymorphisms were associated with specific breast cancer grades (rs11615 with grade 3, rs2228000 and rs50872 with grade 1-2). Two polymorphisms (rs2228001 and rs50872) were associated with the risk of breast cancer with negative lymph node involvement. rs1800975 and rs50872 were associated with the risk of ER^−^ and PR^−^ breast cancer, whereas rs11615 was associated with the risk of ER^+^ and PR^+^ breast cancer. We found that carriers of the T allele of *ERCC1* rs11615, *XPC* rs2228000 and rs50872, particularly in postmenopausal females, have an increased risk of breast cancer.

## INTRODUCTION

Breast cancer is a complex multifactorial disease with unclear etiology. DNA damage and genomic instability, a potential risk of breast cancer, are induced by common environmental factors [2]. However, we are born with a system to protect our genome from DNA damage and correct for damage after it occurs, including nucleotide excision repair (NER), mismatch repair (MMR), bases excision repair (BER), transcription-coupled repair (TCR), and double-strand DNA break repair systems [3].

NER repairs damage introduced by ultraviolet (UV) radiation, products of organic combustion, intrastrand DNA cross-links, heavy metals, and oxidative stress. Several proteins, including ERCC1, XPA, XPB/ERCC3, XPC, XPD/ERCC2, ERCC4/XPF, ERCC5/XPG, and PE/DDB1, are involved in the repair process, maintaining genome integrity to prevent carcinogenesis. The process of NER comprises several distinct steps, including DNA damage recognition, DNA damage demarcation, damaged DNA incision, repair patch synthesis, and ligation. Polymorphisms in NER pathway genes have been associated with increased risk for a number of cancers [4] [5–8].

Breast cancer patients and their relatives tend to have constitutively low NER levels in their peripheral blood lymphocytes [9, 10]. Moreover, polymorphisms in NER pathway genes have been linked to breast cancer risk in studies conducted on patients of some ethnicities. However, the conclusions have been inconsistent [11–14]. Among the Chinese population, studies have reported correlations between polymorphisms in NER pathway genes and breast cancer risk, but to date, there is no systematic investigation on the genetic susceptibility of the NER pathway in breast cancer [15–19]. To provide a more comprehensive understanding of the relationships between specific polymorphisms in the NER pathway genes (Table 1) on the carcinogenesis of breast cancer, we performed a breast cancer risk association study and a meta-analysis.

**Table 1:** Candidate genes and polymorphisms

Gene	rs #	Chromosome	Allele (major/minor)	Position	HWE in controls
*XPA*	rs1800975	9:97697296	G/A	5' non-coding region (-4A/G)	0.118/2.445
*ERCC1*	rs11615	19:45420395	C/T	Exon 4 (Asn118Asn)	0.509/0.436
*XPC*	rs2228000	3:14158387	C/T	Exon 9 (Ala499Val)	0.498/0.460
*XPC*	rs2228001	3:14145949	A/C	Exon 16 (Gln939Lys)	0.267/1.231
*ERCC2/XPD*	rs238406	19:45365051	G/T	Exon 6 (Arg156Arg)	0.766/0.088
*ERCC2/XPD*	rs1799793	19:45364001	G/A	Exon 10 (Asp 312 Asn)	0.101/2.687
*ERCC2/XPD*	rs50872	19:45359191	C/T	Intron 12	0.945/0.005
*ERCC2/XPD*	rs13181	19:45351661	T/A	Exon 23 ( Lys751 Gln)	0.716/0.132
*ERCC2/XPD*	rs3810366	19:45370684	C/G	Promoter (-114)	0.099/2.728
*ERCC4/XPF*	rs1799801	16:13948101	T/C	Exon 11 (Ser835Ser)	0.619/0.247
*ERCC5/XPG*	rs17655	13:102875652	C/G	Exon 15 (His1104Asp)	0.077/3.137

## RESULTS

None of the tested polymorphisms deviated from Hardy-Weinberg equilibrium (HWE) in controls (Table 1). There were no significant differences in the age and menopausal status among cases and controls (Table 2).

**Table 2:** Clinical characteristics of the participants

	Cases, *n* (%)	Controls, *n* (%)	*P* value
**Age (mean ± SD)**	52.85 ± 10.77	52.67 ± 10.78	0.799^*^
**Menopausal status**			0.110
Pre-	206 (45.78)	220 (51.16)	
Post-	244 (54.22)	210 (48.84)	
**Tumor size (T1-T4)**			
T1-T2	312(69.33)		
T3-T4	138(30.67)		
**Tumor grade (G1-G3)**			
G1	86(19.11)		
G2	238(52.89)		
G3	126(28.00)		
**Lymph node involvement**			
Yes	235(52.22)		
No	215(47.78)		
**ER**			
Positive	278(61.78)		
Negative	172(38.22)		
**PR**			
Positive	238(52.89)		
Negative	212(47.11)		
**HER-2**			
Positive	353(78.44)		
Negative	97(21.55)		

* Independent t test applied to age; ER, estrogen receptor; HER-2, human epidermal growth factor receptor-2; PR, progesterone receptor.

The genotype distribution in the two groups and their subgroups of menopausal status are presented in Table 3. The result showed that *ERCC1* rs11615, *XPC* rs2228000, and *ERCC2/XPD* rs50872 carriers have a higher breast cancer risk in the whole study population. Stratified analysis of menopausal status revealed that *XPC* rs2228000 has a higher breast cancer risk in the premenopausal sub-cohort. While in the postmenopausal sub-cohort, *ERCC1* rs11615 and *ERCC2/XPD* rs50872 were associated with increased breast cancer risk. On the contrary, *XPA* rs1800975 and *XPC* rs2228001 were associated with decreased breast cancer risk.

**Table 3:** Distribution of the genotypes in the participants and sub-groups

Genotype	All participants			Premenopause			Postmenopause		
	Ca/Co	OR (95% CI)^*^	*P* value	Ca/Co	OR (95% CI)^#^	*P* value	Ca/Co	OR (95% CI)^#^	*P* value
*XPA* rs1800975									
GG	115/93	Reference		47/55	Reference		68/38	Reference	
GA	235/231	0.82(0.59,1.14)	0.245	106/114	1.06(0.66,1.71)	0.805	129/117	0.63(0.39,1.01)	0.057
AA	100/106	0.77(0.52,1.13)	0.186	53/51	1.21(0.70,2.10)	0.489	47/55	0.48(0.27,0.83)	0.009
GA/AA	335/337	0.81(0.59,1.11)	0.185	159/165	1.11(0.71,1.74)	0.646	176/172	0.59(0.37,0.92)	0.020
Additive model	450/430	0.88(0.73,1.07)	0.198	206/220	1.10(0.84,1.45)	0.488	244/210	0.70(0.53,0.92)	0.012
*ERCC1* rs11615									
CC	230/261	Reference		108/128	Reference		122/133	Reference	
CT	195/151	1.45(1.10,1.92)	0.009	86/86	1.17(0.79,1.74)	0.430	109/65	1.80(1.21,2.68)	0.004
TT	25/18	1.56(0.83,2.94)	0.168	12/6	2.32(0.84,6.41)	0.104	13/12	1.18(0.52,2.69)	0.700
CT/TT	220/169	1.46(1.11,1.91)	0.006	98/92	1.25(0.85,1.83)	0.260	122/77	1.69(1.16,2.47)	0.007
Additive model	450/430	1.36(1.08,1.71)	0.009	206/220	1.29(0.93,1.80)	0.131	244/210	1.42(1.04,1.95)	0.030
*XPC* rs2228000									
CC	201/228	Reference		86/116	Reference		115/112	Reference	
CT	198/174	1.31(0.99,1.73)	0.061	94/85	1.51(1.01,2.26)	0.048	104/89	1.15(0.78,1.69)	0.481
TT	51/28	2.16(1.3,3.57)	0.003	26/19	1.85(0.96,3.57)	0.065	25/9	2.69(1.20,6.02)	0.016
CT/TT	249/212	1.42(1.09,1.86)	0.010	120/104	1.57(1.07,2.30)	0.022	129/98	1.30(0.90,1.88)	0.170
Additive model	450/430	1.39(1.13,1.72)	0.002	206/220	1.41(1.06,1.88)	0.020	244/210	1.37(1.02,1.85)	0.038
*XPC* rs2228001									
AA	193/161	Reference		86/91	Reference		107/70	Reference	
AC	195/213	0.76(0.57,1.01)	0.060	90/100	0.96(0.64,1.45)	0.850	105/113	0.61(0.41,0.91)	0.015
CC	62/56	0.91(0.60,1.38)	0.649	30/29	1.08(0.60,1.96)	0.791	32/27	0.76(0.42,1.38)	0.364
AC/CC	257/269	0.79(0.60,1.04)	0.090	120/129	0.99(0.67,1.45)	0.944	137/140	0.64(0.44,0.94)	0.022
Additive model	450/430	0.90(0.74,1.09)	0.275	206/220	1.02(0.77,1.34)	0.909	244/210	0.79(0.60,1.04)	0.098
*ERCC2/XPD* rs238406									
GG	128/128	Reference		55/62	Reference		73/66	Reference	
GT	227/216	1.05(0.77,1.43)	0.763	108/111	1.09(0.70,1.71)	0.700	119/105	1.01(0.66,1.55)	0.961
TT	95/86	1.12(0.76,1.64)	0.577	43/47	1.05(0.60,1.84)	0.855	52/39	1.19(0.69,2.03)	0.534
GT/TT	322/302	1.07(0.80,1.43)	0.661	151/158	1.08(0.70,1.65)	0.739	171/144	1.06(0.71,1.59)	0.772
Additive model	450/430	1.06(0.87,1.28)	0.583	206/220	1.02(0.78,1.35)	0.866	244/210	1.08(0.83,1.41)	0.546
*ERCC2/XPD* rs1799793									
GG	380/367	Reference		171/192	Reference		209/175	Reference	
GA	69/63	1.05(0.72,1.52)	0.800	35/28	1.41(0.82,2.42)	0.211	34/35	0.80(0.48,1.34)	0.399
AA	1/0	--	--	0/0	--	--	1/0	--	--
GA/AA	70/63	1.06(0.73,1.54)	0.743	35/28	1.41(0.82,2.42)	0.211	35/35	0.82(0.50,1.37)	0.460
Additive model	450/430	1.08(0.75,1.56)	0.683	206/220	1.41(0.82,2.42)	0.211	244/210	0.86(0.52,1.41)	0.543
*ERCC2/XPD* rs50872									
CC	269/290	Reference		130/151	Reference		139/139	Reference	
CT	160/126	1.35(1.01,1.79)	0.044	66/61	1.27(0.83,1.93)	0.270	94/65	1.42(0.96,2.11)	0.081
TT	21/14	1.64(0.82,3.29)	0.165	10/8	1.53(0.58,4.01)	0.388	11/6	1.83(0.66,5.10)	0.245
CT/TT	181/140	1.38(1.04,1.81)	0.024	76/69	1.29(0.86,1.93)	0.212	105/71	1.46(0.99,2.14)	0.054
Additive model	450/430	1.32(1.04,1.67)	0.021	206/220	1.25(0.89,1.75)	0.199	244/210	1.40(1.00,1.95)	0.048
*ERCC2/XPD* rs13181									
TT	361/354	Reference		169/181	Reference		192/173	Reference	
GT	86/73	1.16(0.82,1.63)	0.412	37/38	1.06(0.64,1.75)	0.824	49/35	1.26(0.78,2.03)	0.353
GG	3/3	0.95(0.19,4.74)	0.945	0/1	--	--	3/2	1.40(0.23,8.50)	0.715
GT/GG	89/76	1.15(0.82,1.61)	0.432	37/39	1.03(0.63,1.69)	0.915	52/37	1.27(0.79,2.02)	0.327
Additive model	450/430	1.13(0.82,1.55)	0.472	206/220	0.99(0.61,1.62)	0.980	244/210	1.24(0.81,1.92)	0.326
*ERCC2/XPD* rs3810366									
GG	112/94	Reference		55/56	Reference		57/38	Reference	
CG	234/232	0.84(0.60,1.17)	0.292	107/109	1.00(0.63,1.58)	0.997	127/123	0.69(0.43,1.12)	0.134
CC	104/104	0.83(0.56,1.23)	0.353	44/55	0.80(0.47,1.39)	0.436	60/49	0.84(0.48,1.48)	0.545
CG/CC	338/336	0.84(0.61,1.15)	0.268	151/164	0.93(0.60,1.44)	0.745	187/172	0.74(0.47,1.18)	0.202
Additive model	450/430	0.92(0.76,1.11)	0.375	206/220	0.90(0.69,1.19)	0.462	244/210	0.93(0.70,1.23)	0.598
*ERCC4/XPF* rs1799801									
TT	268/260	Reference		118/136	Reference		150/124	Reference	
CT	157/151	1.01(0.76,1.34)	0.949	78/69	1.31(0.87,1.97)	0.196	79/82	0.79(0.54,1.17)	0.244
CC	25/19	1.31(0.70,2.45)	0.399	10/15	0.76(0.33,1.77)	0.526	15/4	3.03(0.98,9.37)	0.055
CT/CC	182/170	1.04(0.79,1.36)	0.775	88/84	1.22(0.83,1.79)	0.324	94/86	0.90(0.62,1.31)	0.579
Additive model	450/430	1.06(0.85,1.33)	0.593	206/220	1.08(0.79,1.47)	0.647	244/210	1.05(0.76,1.45)	0.763
*ERCC5/XPG* rs17655									
GG	101/107	Reference		48/61	Reference		53/46	Reference	
CG	243/233	1.09(0.79,1.52)	0.588	114/114	1.27(0.80,2.01)	0.311	129/119	0.94(0.59,1.50)	0.796
CC	106/90	1.22(0.82,1.80)	0.332	44/45	1.23(0.70,2.16)	0.471	62/45	1.20(0.69,2.08)	0.524
CG/CC	349/323	1.12(0.82,1.54)	0.464	158/159	1.26(0.81,1.95)	0.308	191/164	1.00(0.64,1.57)	1.000
Additive model	450/430	1.11(0.91,1.35)	0.307	206/220	1.12(0.85,1.48)	0.433	244/210	1.10(0.83,1.44)	0.509

* Adjusted by age and menopausal status;

# Adjusted by age; Ca, case; Co, control.

Based on the observed significant associations, we then performed stratified analysis based on pathological characteristics of the breast cancer (tumor size, lymph node involvement) and expression of specific proteins in tumor tissue (PR, ER, and HER-2). Tumor size (T3-T4) was associated with all polymorphisms of interest except for *XPA* rs1800975. In addition, *ERCC1* rs11615 carriers have a high risk of breast cancer with grade 3, while *XPC* rs2228000 and *ERCC2/XPD* rs50872 are linked to a high risk for breast cancer with grades 1 and 2, respectively. For the lymph node involvement subgroup, *XPC* rs2228001 and *ERCC2/XPD* rs50872 carriers have a high risk of breast cancer with negative lymph node involvement. While *ERCC1* rs11615 and *XPC* rs2228000, were significantly associated with both negative and positive lymph node involvement subgroups (Table 4).

**Table 4:** Polymorphisms on breast cancer risk by pathological characteristics of tumor

Genotype	Co	Tumor size (T1-T2)			Tumor size (T3-T4)			Grade (G1-G2)			Grade (G3)			Lymph node involvement(Negative)			Lymph node involvement (Positive)		
		Ca	OR (95% CI)^*^	*P* value	Ca	OR (95% CI)^*^	*P* value	Ca	OR (95% CI)^*^	*P* value	Ca	OR (95% CI)^*^	*P* value	Ca	OR(95% CI)^*^	*P* value	Ca	OR(95% CI)^*^	*P* value
*XPA* rs1800975																			
GG	93	77	Reference		38	Reference		80	Reference		35	Reference		57	Reference		58	Reference	
GA	231	167	0.88(0.61,1.26)	0.482	68	0.71(0.45,1.14)	0.158	177	0.89(0.62,1.28)	0.535	58	0.65(0.40,1.06)	0.087	111	0.78(0.52,1.16)	0.216	124	0.86(0.58,1.29)	0.470
AA	106	68	0.78(0.51,1.21)	0.267	32	0.74(0.42,1.27)	0.271	67	0.74(0.48,1.14)	0.176	33	0.80(0.46,1.40)	0.432	47	0.73(0.45,1.17)	0.188	53	0.80(0.50,1.28)	0.353
GA/AA	337	235	0.85(0.60,1.20)	0.359	100	0.73(0.47,1.13)	0.155	244	0.85(0.60,1.20)	0.350	91	0.71(0.45,1.12)	0.139	158	0.77(0.52,1.12)	0.168	177	0.85(0.58,1.24)	0.395
*ERCC1* rs11615																			
CC	261	163	Reference		67	Reference		176	Reference		54	Reference		117	Reference		113	Reference	
TC	151	128	1.36(1.00,1.85)	0.054	67	1.70(1.15,2.53)	0.008	128	1.24(0.91,1.68)	0.171	67	2.19(1.45,3.32)	0.000	81	1.18(0.83,1.68)	0.348	114	1.75(1.26,2.44)	0.001
TT	18	21	1.85(0.95,3.57)	0.069	4	0.90(0.29,2.76)	0.850	20	1.64(0.84,3.19)	0.147	5	1.32(0.47,3.74)	0.595	17	2.12(1.05,4.27)	0.035	8	1.00(0.42,2.38)	0.999
TC/TT	169	149	1.40(1.04,1.88)	0.028	71	1.61(1.09,2.37)	0.016	148	1.27(0.95,1.71)	0.109	72	2.07(1.38,3.11)	0.000	98	1.27(0.91,1.78)	0.154	122	1.65(1.20,2.28)	0.002
*XPC* rs2228000																			
CC	228	145	Reference		56	Reference		141	Reference		60	Reference		98	Reference		103	Reference	
CT	174	139	1.28(0.94,1.74)	0.119	59	1.39(0.91,2.10)	0.124	149	1.41(1.04,1.91)	0.028	49	1.06(0.69,1.63)	0.782	91	1.24(0.88,1.76)	0.226	107	1.36(0.97,1.90)	0.074
TT	28	28	1.69(0.95,2.99)	0.074	23	3.43(1.83,6.44)	0.000	34	2.08(1.20,3.60)	0.009	17	2.42(1.24,4.75)	0.010	26	2.39(1.32,4.34)	0.004	25	2.02(1.12,3.64)	0.020
CT/TT	202	167	1.33(0.99,1.78)	0.058	82	1.67(1.13,2.46)	0.010	183	1.49(1.12,2.00)	0.007	66	1.25(0.84,1.86)	0.280	117	1.38(0.99,1.92)	0.056	132	1.45(1.05,2.00)	0.023
*XPC* rs2228001																			
AA	161	127	Reference		66	Reference		139	Reference		54	Reference		96	Reference		97	Reference	
AC	213	139	0.81(0.59,1.12)	0.205	56	0.64(0.42,0.96)	0.033	142	0.77(0.56,1.05)	0.095	53	0.73(0.48,1.13)	0.160	86	0.67(0.47,0.95)	0.026	109	0.85(0.60,1.19)	0.341
CC	56	46	1.02(0.64,1.61)	0.940	16	0.68(0.36,1.27)	0.225	43	0.87(0.55,1.38)	0.548	19	1.00(0.54,1.85)	0.996	33	0.97(0.59,1.61)	0.916	29	0.83(0.49,1.40)	0.483
AC/CC	269	185	0.86(0.64,1.16)	0.319	72	0.65(0.44,0.95)	0.027	185	0.79(0.59,1.06)	0.115	72	0.79(0.53,1.18)	0.252	119	0.73(0.52,1.02)	0.064	138	0.85(0.61,1.17)	0.317
*ERCC2/XPD* rs50872																			
CC	290	192	Reference		77	Reference		195	Reference		74	Reference		126	Reference		143	Reference	
CT	126	105	1.24(0.90,1.70)	0.186	55	1.62(1.08,2.43)	0.020	113	1.31(0.96,1.80)	0.089	47	1.45(0.95,2.21)	0.087	79	1.42(1.00,2.03)	0.049	81	1.29(0.91,1.82)	0.154
TT	14	15	1.68(0.79,3.57)	0.180	6	1.63(0.60,4.37)	0.336	16	1.77(0.84,3.72)	0.134	5	1.41(0.49,4.08)	0.522	10	1.69(0.73,3.91)	0.223	11	1.62(0.72,3.68)	0.247
CT/TT	140	120	1.28(0.94,1.74)	0.113	61	1.62(1.09,2.39)	0.017	129	1.36(1.01,1.84)	0.046	52	1.44(0.96,2.17)	0.082	89	1.45(1.03,2.04)	0.032	92	1.32(0.95,1.84)	0.104

* Adjusted by age and menopausal status; Ca, case; Co, control.

For tumor tissue characteristics, *XPA* rs1800975 and *ERCC2/XPD* rs50872 carriers have a high risk of breast cancer with negative expression of ER and PR. While *ERCC1* rs11615 have a high risk of ER+ and PR+ breast cancer and PR. The susceptibility of *XPC* rs2228000 to breast cancer risk was observed in both subgroups; however, there was no significant association for *XPC* rs2228001 in any subgroup (Table 5).

**Table 5:** Effects of five SNPs on breast cancer risk as stratified by expression of ER, PR, and HER-2

Genotype	Co	ER (-)			ER (+)			PR (-)			PR (+)			HER-2 (-)			HER-2 (+)		
		Ca	OR(95% CI)^*^	*P* value	Ca	OR(95% CI)^*^	*P* value	Ca	OR(95% CI)^*^	*P* value	Ca	OR(95% CI)^*^	*P* value	Ca	OR(95% CI)^*^	*P* value	Ca	OR(95% CI)^*^	*P* value
*XPA* rs1800975																			
GG	93	51	Reference		64	Reference		61	Reference		54	Reference		27	Reference		88	Reference	
GA	231	86	0.67(0.44,1.03)	0.067	149	0.93(0.64,1.36)	0.718	109	0.72(0.49,1.08)	0.113	126	0.93(0.62,1.39)	0.720	46	0.69(0.41,1.18)	0.177	189	0.86(0.60,1.22)	0.399
AA	106	35	0.60(0.36,1.01)	0.055	65	0.89(0.57,1.39)	0.602	42	0.61(0.37,0.99)	0.044	58	0.94(0.59,1.49)	0.786	24	0.78(0.42,1.45)	0.432	76	0.76(0.50,1.15)	0.196
GA/AA	337	121	0.65(0.44,0.98)	0.038	214	0.92(0.64,1.33)	0.670	151	0.69(0.47,1.00)	0.053	184	0.94(0.64,1.38)	0.747	70	0.73(0.44,1.20)	0.212	265	0.83(0.60,1.16)	0.284
*ERCC1* rs11615																			
CC	261	98	Reference		132	Reference		119	Reference		111	Reference		47	Reference		183	Reference	
TC	151	70	1.23(0.85,1.78)	0.276	125	1.63(1.18,2.24)	0.003	82	1.16(0.82,1.64)	0.414	113	1.78(1.28,2.48)	0.001	48	1.73(1.10,2.72)	0.018	147	1.38(1.03,1.87)	0.034
TT	18	4	0.58(0.19,1.76)	0.333	21	2.28(1.17,4.44)	0.015	11	1.34(0.61,2.94)	0.463	14	1.81(0.87,3.77)	0.114	2	0.66(0.15,2.95)	0.585	23	1.80(0.94,3.44)	0.075
TC/TT	169	74	1.16(0.81,1.66)	0.432	146	1.68(1.24,2.29)	0.001	93	1.17(0.84,1.64)	0.352	127	1.76(1.28,2.43)	0.001	50	1.61(1.03,2.51)	0.037	170	1.41(1.06,1.88)	0.018
*XPC* rs2228000																			
CC	228	84	Reference		117	Reference		96	Reference		105	Reference		41	Reference		160	Reference	
CT	174	67	1.06(0.72,1.54)	0.773	131	1.48(1.08,2.04)	0.016	88	1.23(0.86,1.75)	0.257	110	1.38(0.99,1.92)	0.059	41	1.32(0.82,2.12)	0.257	157	1.30(0.97,1.75)	0.082
TT	28	21	2.27(1.20,4.26)	0.011	30	2.15(1.22,3.78)	0.008	28	2.62(1.45,4.73)	0.001	23	1.83(1.00,3.34)	0.049	15	3.09(1.50,6.36)	0.002	36	1.93(1.12,3.31)	0.017
CT/TT	202	88	1.21(0.85,1.72)	0.299	161	1.58(1.16,2.14)	0.004	116	1.41(1.01,1.96)	0.045	133	1.44(1.05,1.98)	0.026	56	1.55(0.99,2.42)	0.055	193	1.39(1.04,1.84)	0.025
*XPC* rs2228001																			
AA	161	72	Reference		121	Reference		94	Reference		99	Reference		43	Reference		150	Reference	
AC	213	79	0.81(0.55,1.19)	0.277	116	0.72(0.52,1.00)	0.051	90	0.71(0.49,1.01)	0.055	105	0.80(0.57,1.13)	0.202	42	0.74(0.46,1.20)	0.220	153	0.76(0.56,1.04)	0.085
CC	56	21	0.80(0.45,1.42)	0.444	41	0.97(0.60,1.55)	0.894	28	0.81(0.48,1.37)	0.436	34	0.99(0.60,1.62)	0.958	12	0.77(0.38,1.58)	0.482	50	0.94(0.60,1.47)	0.790
AC/CC	269	100	0.81(0.56,1.16)	0.251	157	0.77(0.57,1.05)	0.098	118	0.73(0.52,1.02)	0.066	139	0.84(0.61,1.16)	0.284	54	0.75(0.48,1.18)	0.213	203	0.80(0.60,1.07)	0.133
*ERCC2/XPD* rs50872																			
CC	290	89	Reference		180	Reference		113	Reference		156	Reference		58	Reference		211	Reference	
CT	126	73	1.86(1.28,2.71)	0.001	87	1.10(0.79,1.53)	0.588	85	1.71(1.20,2.43)	0.003	75	1.09(0.77,1.55)	0.621	36	1.43(0.89,2.28)	0.136	124	1.33(0.98,1.81)	0.069
TT	14	10	2.44(1.04,5.73)	0.040	11	1.27(0.56,2.86)	0.565	14	2.61(1.20,5.68)	0.016	7	0.93(0.37,2.36)	0.882	3	1.08(0.30,3.90)	0.905	18	1.80(0.88,3.72)	0.110
CT/TT	140	83	1.92(1.34,2.76)	0.000	98	1.11(0.81,1.53)	0.518	99	1.80(1.28,2.52)	0.001	82	1.08(0.77,1.51)	0.658	39	1.39(0.88,2.19)	0.157	142	1.38(1.03,1.85)	0.033

* Adjusted by age and menopausal status; Ca, case; Co, control.

For *ERCC2/XPD* rs238406, rs1799793, rs13181, rs3810366, *ERCC4/XPF* rs1799801, *ERCC5/XPG* rs17655, no significant association was found (Table 3).

To confirm the results of our case study, we performed a meta-analysis involving *XPC* rs2228000, rs2228001, *XPA* rs1800975, and *ERCC1* rs11615 (Table 6). We identified 14 studies for the meta-analysis according to the inclusion criteria. The characteristics of the selected studies are presented in Supplemental Table S1. The allele frequencies of the four polymorphisms in Asian and Caucasian populations are shown in Supplemental Table S2, indicating the allele frequencies of this study were consistent with those of the pooled data.

**Table 6:** Meta-analysis of the XPC rs2228000, rs2228001, XPA rs1800975 and ERCC1 rs11615 polymorphism on breast cancer risk

Variables	Cases/controls	Homozygote vs. wild type			Heterozygote vs. wild type			Dominant model			Recessive model^d^		
		OR(95% CI)	*P* value	*P_h_^b^*	OR(95% CI)	*P* value	*P_h_^b^*	OR (95% CI)	*P* value	*P_h_^b^*	OR(95% CI)	*P* value	*P_h_^b^*
*XPC* rs2228000													
Total	3897/4877^a^	1.28(1.08-1.52)	0.004	0.228	1.01(0.92,1.10)	0.867	0.291	1.02(0.89,1.17)^c^	0.766	0.030	1.25(1.06, 1.47)	0.008	0.521
**Ethnicities**													
Caucasian	570/676	0.95(0.59,1.51)	0.811	0.979	0.86(0.68,1.08)	0.196	0.983	0.87(0.69,1.09)	0.219	0.991	1.01(0.64,1.59)	0.981	0.990
Asian	1068/1052	1.73(1.30,2.31)	0.000	0.384	1.26(1.05,1.51)	0.015	0.803	1.37(1.15,1.64)	0.000	0.475	1.52(1.16,1.99)	0.002	0.349
Other	2259/3149	1.13(0.89,1.43)	0.318	0.748	0.97(0.86,1.08)	0.520	0.809	0.97(0.88,1.08)	0.597	0.557	1.14(0.90,1.43)	0.287	0.674
**Source of control**													
PB	2364/3220	1.37(1.11,1.68)	0.003	0.107	1.09(0.97,1.22)	0.148	0.198	1.15(0.94,1.42)^c^	0.171	0.033	1.29(1.06,1.57)	0.010	0.266
HB	1533/1657	1.12(0.82,1.51)	0.485	0.642	0.91(0.79,1.04)	0.149	0.931	0.91(0.80,1.04)	0.164	0.717	1.15(0.86,1.56)	0.349	0.656
*XPC* rs2228001													
Total	6176/6955	0.99(0.89,1.10)	0.850	0.343	0.97(0.90,1.05)	0.430	0.206	0.97(0.91,1.05)	0.470	0.180	1.01(0.91,1.11)	0.869	0.444
**Ethnicities**													
Caucasian	1714/1613	0.85(0.70,1.05)	0.369	0.369	0.91(0.78,1.05)	0.194	0.608	0.89(0.78,1.03)	0.112	0.462	0.90(0.75,1.09)	0.279	0.485
African	814/753	0.90(0.60,1.35)	0.649	0.649	0.94(0.77,1.16)	0.567	0.308	0.94(0.77,1.14)	0.512	0.420	0.93(0.63,1.37)	0.716	0.513
Asian	1068/1052	1.14(0.87,1.49)	0.196	0.196	1.01(0.59,1.75)^c^	0.962	0.004	1.04(0.63,1.71)^c^	0.894	0.005	1.10(0.86,1.42)	0.446	0.826
Other	2580/3537	1.04(0.89,1.22)	0.298	0.298	0.99(0.88,1.10)	0.810	0.792	1.00(0.90,1.11)	0.998	0.891	1.06(0.92,1.21)	0.453	0.130
**Source of control**													
PB	4587/5222	0.95(0.84,1.08)	0.441	0.377	0.95(0.87,1.04)	0.258	0.081	0.95(0.88,1.03)^b^	0.230	0.056	0.98(0.87,1.10)	0.691	0.795
HB	1589/1733	1.10(0.89,1.35)	0.371	0.336	1.03(0.89,1.20)	0.700	0.738	1.05(0.91,1.21)	0.523	0.971	1.09(0.91,1.32)	0.346	0.122
*XPA* rs1800975													
Total	2619/2663	0.92(0.65,1.31)	0.649	0.003	1.07(0.78,1.48)	0.663	0.001	1.03(0.74,1.42)	0.873	0.000	0.94(0.84,1.06)	0.303	0.190
Ethnicities													
Asian	1407/1409	0.82(0.54,1.26)	0.372	0.018	0.95(0.60,1.51)	0.841	0.002	0.91(0.58,1.43)	0.686	0.001	0.83(0.70,0.99)	0.039	0.539
Other	1212/1254	1.22(0.94,1.59)	0.129	0.255	1.30(1.01,1.66)	0.039	0.821	1.27(1.01,1.60)	0.046	0.795	1.05(0.89,1.23)	0.585	0.239
*ERCC1* rs11615													
Total	1012/1035	1.56(1.17,2.09)	0.003	0.717	1.31(1.09,1.58)	0.005	0.462	1.38(1.15,1.64)	0.000	0.781	1.44(1.10,1.90)	0.009	0.634

a rs2228000 cases/controls are not include the studies of Smith(b) and Perez-Mayoral

b Ph value of Q-test for heterogeneity test.

c Random-effects model was used when a P value < 0.05 for heterogeneity test; otherwise, fixed-effects model was used.

d Available data by Tatemichi et al was used in stratified analyses by cancer type, ethnicity and source of control for the recessive model comparison. PB: population based control studies; HB: hospital based control studies.

Pooled results suggested that *XPC* rs2228000 TT was associated with increased breast cancer risk. In addition, in the Asian population subgroup, *XPC* rs2228000 TT genotype was a risk factor for breast cancer (Table 6). Similarly, in the population-based studies subgroup, *XPC* rs2228000 TT genotype was correlated with an increased risk of breast cancer (Table 6). For *XPC* rs2228001, no significant association was found by pooled or subgroup analysis.

For *XPA* rs1800975, there were no significant associations with breast cancer risk in the pooled results or the Asian population subgroup; however, in the other ethnic population subgroup, a weak but significant association with increased breast cancer was observed in both the co-dominant and dominant models (Table 6). For *ERCC1* rs11615, the pooled results indicated that TT and TT/CT genotype were associated with increased breast cancer risk (Table 6).

## DISCUSSION

This case-control association study revealed that *ERCC1* rs11615 (T allele), *XPC* rs2228000 (T allele) and *ERCC2/XPD* rs50872 (T allele) were associated with increased breast cancer risk. Besides, *ERCC1* rs11615 (T allele), and *ERCC2/XPD* rs50872 (T allele) were associated with postmenopausal breast cancer, while *XPC* rs2228000 (T allele) was associated with premenopausal breast cancer.

The *XPC* gene encodes a 940 amino acid protein that forms an XPC-RAD23B complex with RAD23B [20]. *XPC* rs2228000 is a C-to-T transition causing a substitution in codon 499 in exon 8 that changes alanine to valine in the interaction domain of *XPC* with *hHRAD23*. Consistent with previous reports which linked the TT genotype with lower DNA repair capacity (DRC), [21] this study found that T allele (CT/TT) carriers have a higher breast cancer risk. An independent study reported that presence of the *XPC* rs2228000 T allele (CT or TT genotype) was associated with estrogen receptor positive breast cancer [22]. In all, these studies suggest that patients harboring the *XPC* rs2228000 T allele have a higher risk of breast cancer. Furthermore, the significance of this association was confirmed by the result of the meta-analysis.

Our study revealed that *XPC* rs2228001 was not a risk factor for breast cancer, and this was confirmed by our meta-analysis. Our subgroup analysis revealed that postmenopausal females with AC or AC/CC genotype have a lower breast cancer risk. To our knowledge, this is the first study reporting these results, which should be verified by further work.

Postmenopausal females with *XPA* rs1800975 carrying one or two A alleles have a higher breast cancer risk than those with GG genotype, consistent with reports on populations of northern Chinese [23] and South Korean women [24]. On the other hand, a functional study showed that the *XPA* rs1800975 G allele increased promoter activity [25] leading to increased *XPA* protein concentration [26]. Therefore, *XPA* rs1800975 AA genotype was recognized as a risk factor for lung cancer [27]. It is interesting to see contrasting results among different kinds of cancer, suggesting the susceptibility of *XPA* rs1800975 to cancer risk may be dependent on cancer type.

*ERCC1* variant rs11615 (C19007T) is a C>T synonymous polymorphism in exon 4 (Asn118Asn), converting a high-usage codon AAC to a low-usage codon AAU. This case-control study revealed the susceptibility of carriers of *ERCC1* variant rs11615 to increased risk of breast cancer, consistent with previous observations that *ERCC1* rs11615 was associated with reduced mRNA [28] and protein [29] expression levels, and consequently impaired DNA repair capacity [28]. Therefore, *ERCC1* rs11615 T allele carriers (CT/TT) exhibited reduced *ERCC1* expression and higher breast cancer risk, which was consistent with our results. This association was supported by the pooled results of this meta-analysis and the study carried out on a population in China [30]. Additionally, in our study the increased risk of breast cancer linked to *ERCC1* rs11615 more prominent in postmenopausal females and patients with positive expression of PR and ER, indicating the risk conveyed by this polymorphism to breast cancer in menopausal females [30].

*ERCC2/XPD* rs50872 is a C/T polymorphism in intron 4 of *XPD*. This case-control study linked *ERCC2/XPD* rs50872 to increased breast cancer risk and showed the polymorphism was more prevalent in the patients with tumor size T3-T4, negative lymph node involvement and patients with ER^−^ and PR^−^ expression, which was consistent with the conclusions in a South Korean population [24].

Some limitations of this study should be noted. First, the relatively small sample size may limit the statistical power to find differences among groups and therefore some associations may be missed, particularly in the multiple stratified analyses. Therefore, we carried out a meta-analysis to confirm the results of the case-control study. Second, several potential environmental factors, such as occupational exposure and diet, were not included in this study, which may influence breast cancer risk. Third, patients’ clinical outcomes were not traced for the analysis of the predictive value of polymorphisms in the NER pathway. Finally, the polymorphisms included in this study were still limited, and these polymorphisms were selected based on previous knowledge of their potential functional roles in the occurrence of cancers. Analysis of a wider range of polymorphisms would provide more complete information about the associations of NER genes and breast cancer risk.

In conclusion, our study deduced that *ERCC1* rs11615 (CT or CT/TT), *XPC* rs2228000 (TT or CT/TT) and rs50872 (CT or CT/TT) were risk factors associated with increased breast cancer incidence, especially in postmenopausal women. The risk conferred by polymorphisms in NER pathway genes for breast cancer among females with different menopausal status should be evaluated in a larger cohort study.

## MATERIALS AND METHODS

### Study subjects

For the case-control association study, from January 2008 to January 2015 in Nanjing First Hospital, Nanjing Medical University, China, we enrolled 450 female patients histologically diagnosed with breast cancer, and 430 age-matched healthy females, who visited the same hospital for routine physical examination, were enrolled as non-cancer controls. All participants were from the same geographic region. The clinical characteristics of each subject, including smoking, drinking, and other cancer history, were collected via a questionnaire and written informed consents were obtained from all participants. Participants were enrolled in this study with no limitation for the smoking and drinking or not, and finally, there were less than ten individuals with a history of smoking and drinking, which may be attributed to the lifestyle of Chinese females. We excluded these samples as unrepresentative of the population before genotyping. The protocol of this study was approved by the Institutional Review Board of Nanjing First Hospital.

### Genotyping of polymorphisms

Genotyping was performed as we described previously [31, 32]. The genotyping for all the polymorphisms was performed by Sequenom MassARRAY RS1000 according to the standard protocol. Multiplexed SNP MassEXTENDED assay was designed by Sequenom MassARRAY Assay Design 3.0 Software [33]. Finally, data management and analysis were performed by Sequenom Typer 4.0 Software [33, 34].

### Immunohistochemistry (IHC) assay

The expression of ER, PR, and HER-2 in paraffin-embedded tumor tissue samples was evaluated by immunohistochemistry (IHC) assay, as we described previously [31, 32].

### Meta-analysis of polymorphisms in *XPA* (rs1800975), *XPC* (rs2228000, rs2228001), and *ERCC1* (rs11615)

Meta-analysis was performed to confirm the polymorphisms identified as breast cancer risk factors by our case-control study. Four polymorphisms (*XPA* (rs1800975), *XPC* (rs2228000, rs2228001), and *ERCC1* (rs11615)) were evaluated for breast cancer risk using pooled data from this study and available published studies. The *ERCC2/XPD* rs50873 was ruled out for lack of available published data.

To identify relevant studies, we searched PubMed and Embase databases using the keywords ‘XPA,' ‘XPC’ or ‘ERCC1’, ‘polymorphism,' and ‘breast cancer’ (updated to March 31, 2016). The papers were limited to studies on human subjects and published in English. In addition, references listed in any reviews were manually searched to ensure all relevant studies were included. Then, we evaluated the collected publications by screening the titles and abstracts. All studies which matched the following inclusion criteria were retrieved: (i) evaluated at least one of these four polymorphisms (*XPC* rs2228000, rs2228001, *XPA* rs1800975, and *ERCC1* rs11615) and risk of breast cancer; (ii) from a case-control association study; and (iii) with available genotype frequencies.

All data complying with the selection criteria were extracted by two authors (B. H., and T. X.), independently. For each study, the following characteristics were extracted: the first author's last name, country of origin, patient ethnicity, the number of genotyped cases and controls, and the result of this case-control study was also applied for the meta-analysis. For the stratified analysis, subgroup analysis was performed according to ethnicity, which were categorized as Caucasian, Asian, and other; those with mixed ethnicities were categorized as others. In addition, subgroup analysis based on the origin of controls was also applied according to the participants of enrolled studies from population or hospital.

### Statistical analysis

For the case-control association study, the statistical analysis of genotype distribution was performed by *χ*^2^ test. The risk of polymorphisms was evaluated by odds ratios (OR) and 95% confidence intervals (CIs), which were calculated using a logistic regression model. *P* value < 0.05 was considered to have statistically significant difference. Software SPSS 11.0 for Windows (SPSS, Chicago, IL, USA) was used for the statistics.

For the meta-analysis, the overall risk associated with a polymorphism to breast cancer was measured by ORs with 95% CIs based on different genetic models [Rare allele homozygote (RR), heterozygous (WR), and RR+WR *vs*. wild-type homozygote (WW) genotypes]. Stratified analyses were performed by ethnicity. The *Z* test was performed to calculate the pooled OR, and a *P* value < 0.05 was considered as significant. The *χ*^2^ based Q statistical test was used to evaluated the heterogeneity across the enrolled studies [36], and a *P* value of heterogeneity (*P*_h_) < 0.05 was considered significant. The random-effects model was used when there was marked heterogeneity across all the studies; otherwise, the fixed-effects model was used [37]. All statistical tests for this meta-analysis were performed with STATA version 10.0 (Stata Corporation College Station, TX, USA).

## SUPPLEMENTARY MATERIALS FIGURES AND TABLES




