# Supplementary material for: Nucleotide excision repair pathway gene polymorphisms are linked to breast cancer risk in a Chinese population
Source: Oncotarget. 2016 Oct 19;7(51):84872–82. doi: 10.18632/oncotarget.12744 (PMC5356705; doi:10.18632/oncotarget.12744)
Supplement: Supplementary file 1 [file oncotarget-07-84872-s001.pdf]

Nucleotide excision repair pathway gene polymorphisms are linked to breast cancer risk in a Chinese population

SUPPLEMENTARY TABLES

Supplemental Table S1: Characteristics of enrolled articles for the meta-analysis

	First author	Year	Country	Ethnicity	Source of controls	Genotyping method	Cases	Controls	Polymorphisms
1	Forsti	2004	Poland	Caucasian	PB	PCR-RFLP	166	203	rs2228001
2	Mechanic(a)	2006	USA	African	PB	TaqMan	761	679	rs2228001
3	Mechanic(b)	2006	USA	Caucasian	PB	TaqMan	1267	1123	rs2228001
4	Shen	2006	USA	Mixed	HB	TaqMan	155	150	rs2228000, rs2228001, rs1800975
5	Crew	2007	USA	Mixed	PB	Taqman	1057	1104	rs1800975
6	Jorgensen	2007	USA	Caucasian	PB	TaqMan	281	287	rs2228000,rs2228001
7	Shen	2008	USA	Mixed	HB	Fluorescence polarization	1060	1103	rs2228000,rs2228001
8	Smith(a)	2008	USA	Caucasian	HB	MassARRAY	321	406	rs2228000,rs2228001
9	Smith(b)	2008	USA	African	HB	MassARRAY	53	74	rs2228000,rs2228001
10	Roberts	2011	USA	Mixed	PB	Mass spectrometry	1044	1878	rs2228000,rs2228001
11	Han	2012	South Korea	Asian	PB	Illumina BeadStation 500 GX	361	346	rs1800975
12	Yang	2012	China	Asian	PB	PCR-RFLP	618	622	rs2228000,rs2228001
13	Perez-Mayoral	2013	Puerto Rico	Mixed	HB	TaqMan	355	178	rs2228000
14	Yang	2013	China	Asian	PB	MassARRAY	461	504	rs11615
15	Ding	2015	China	Asian	PB	PCR-LDR	596	633	rs1800975
16	Zhu	2015	China	Asian	PB	PCR-RFLP	101	101	rs11615

Supplemental Table S2: Minor allele frequencies (MAF) of control in this study and meta-analysis

Variables	All enrolled studies		Caucasian		Asian		This study
	Number of studies	MAF	Number of studies	MAF	Number of studies	MAF	MAF
rs2228000	8	0.265	2	0.253	1	0.371	0.267
rs2228001	10	0.383	3	0.403	1	0.355	0.378
rs1800975	4	0.575	-	-	2	0.511	0.485
Rs11615	2	0.333	-	-	2	0.333	0.480
