# Supplementary material for: Nucleotide excision repair pathway gene polymorphisms are linked to breast cancer risk in a Chinese population
Source: Oncotarget. 2016 Oct 19;7(51):84872–82. doi: 10.18632/oncotarget.12744 (PMC5356705; doi:10.18632/oncotarget.12744)
Supplement: Supplementary file 2 [file oncotarget-07-84872-s002.docx]

**Table 4. Polymorphisms on Breast Cancer Risk by Pathological Characteristics of Tumor**

| **Genotype** | **Co** | **Tumor size (T1-T2)** | | | **Tumor size (T3-T4)** | | | **Grade (G1-G2)** | | | **Grade (G3)** | | | **Lymph node involvement (Negative)** | | | **Lymph node involvement**  **(Positive)** | | |
| --- | --- | --- | --- | --- | --- | --- | --- | --- | --- | --- | --- | --- | --- | --- | --- | --- | --- | --- | --- |
|  |  | **Ca** | **OR (95% CI)*** | ***P* value** | **Ca** | **OR (95% CI)*** | ***P* value** | **Ca** | **OR (95% CI)*** | ***P* value** | **Ca** | **OR (95% CI)*** | ***P* value** | **Ca** | **OR(95% CI)*** | ***P* value** | **Ca** | **OR(95% CI)*** | ***P* value** |
| *XPA* rs1800975 | | | | | | | | | | | | | | | | | | | |
| GG | 93 | 77 | Reference |  | 38 | Reference |  | 80 | Reference |  | 35 | Reference |  | 57 | Reference |  | 58 | Reference |  |
| GA | 231 | 167 | 0.88(0.61,1.26) | 0.482 | 68 | 0.71(0.45,1.14) | 0.158 | 177 | 0.89(0.62,1.28) | 0.535 | 58 | 0.65(0.40,1.06) | 0.087 | 111 | 0.78(0.52,1.16) | 0.216 | 124 | 0.86(0.58,1.29) | 0.470 |
| AA | 106 | 68 | 0.78(0.51,1.21) | 0.267 | 32 | 0.74(0.42,1.27) | 0.271 | 67 | 0.74(0.48,1.14) | 0.176 | 33 | 0.80(0.46,1.40) | 0.432 | 47 | 0.73(0.45,1.17) | 0.188 | 53 | 0.80(0.50,1.28) | 0.353 |
| GA/AA | 337 | 235 | 0.85(0.60,1.20) | 0.359 | 100 | 0.73(0.47,1.13) | 0.155 | 244 | 0.85(0.60,1.20) | 0.350 | 91 | 0.71(0.45,1.12) | 0.139 | 158 | 0.77(0.52,1.12) | 0.168 | 177 | 0.85(0.58,1.24) | 0.395 |
| *ERCC1*rs11615 | | | | | | | | | | | | | | | | | | | |
| CC | 261 | 163 | Reference |  | 67 | Reference |  | 176 | Reference |  | 54 | Reference |  | 117 | Reference |  | 113 | Reference |  |
| TC | 151 | 128 | 1.36(1.00,1.85) | 0.054 | 67 | 1.70(1.15,2.53) | 0.008 | 128 | 1.24(0.91,1.68) | 0.171 | 67 | 2.19(1.45,3.32) | 0.000 | 81 | 1.18(0.83,1.68) | 0.348 | 114 | 1.75(1.26,2.44) | 0.001 |
| TT | 18 | 21 | 1.85(0.95,3.57) | 0.069 | 4 | 0.90(0.29,2.76) | 0.850 | 20 | 1.64(0.84,3.19) | 0.147 | 5 | 1.32(0.47,3.74) | 0.595 | 17 | 2.12(1.05,4.27) | 0.035 | 8 | 1.00(0.42,2.38) | 0.999 |
| TC/TT | 169 | 149 | 1.40(1.04,1.88) | 0.028 | 71 | 1.61(1.09,2.37) | 0.016 | 148 | 1.27(0.95,1.71) | 0.109 | 72 | 2.07(1.38,3.11) | 0.000 | 98 | 1.27(0.91,1.78) | 0.154 | 122 | 1.65(1.20,2.28) | 0.002 |
| *XPC* rs2228000 | | | | | | | | | | | | | | | | | | | |
| CC | 228 | 145 | Reference |  | 56 | Reference |  | 141 | Reference |  | 60 | Reference |  | 98 | Reference |  | 103 | Reference |  |
| CT | 174 | 139 | 1.28(0.94,1.74) | 0.119 | 59 | 1.39(0.91,2.10) | 0.124 | 149 | 1.41(1.04,1.91) | 0.028 | 49 | 1.06(0.69,1.63) | 0.782 | 91 | 1.24(0.88,1.76) | 0.226 | 107 | 1.36(0.97,1.90) | 0.074 |
| TT | 28 | 28 | 1.69(0.95,2.99) | 0.074 | 23 | 3.43(1.83,6.44) | 0.000 | 34 | 2.08(1.20,3.60) | 0.009 | 17 | 2.42(1.24,4.75) | 0.010 | 26 | 2.39(1.32,4.34) | 0.004 | 25 | 2.02(1.12,3.64) | 0.020 |
| CT/TT | 202 | 167 | 1.33(0.99,1.78) | 0.058 | 82 | 1.67(1.13,2.46) | 0.010 | 183 | 1.49(1.12,2.00) | 0.007 | 66 | 1.25(0.84,1.86) | 0.280 | 117 | 1.38(0.99,1.92) | 0.056 | 132 | 1.45(1.05,2.00) | 0.023 |
| *XPC* rs2228001 | | | | | | | | | | | | | | | | | | | |
| AA | 161 | 127 | Reference |  | 66 | Reference |  | 139 | Reference |  | 54 | Reference |  | 96 | Reference |  | 97 | Reference |  |
| AC | 213 | 139 | 0.81(0.59,1.12) | 0.205 | 56 | 0.64(0.42,0.96) | 0.033 | 142 | 0.77(0.56,1.05) | 0.095 | 53 | 0.73(0.48,1.13) | 0.160 | 86 | 0.67(0.47,0.95) | 0.026 | 109 | 0.85(0.60,1.19) | 0.341 |
| CC | 56 | 46 | 1.02(0.64,1.61) | 0.940 | 16 | 0.68(0.36,1.27) | 0.225 | 43 | 0.87(0.55,1.38) | 0.548 | 19 | 1.00(0.54,1.85) | 0.996 | 33 | 0.97(0.59,1.61) | 0.916 | 29 | 0.83(0.49,1.40) | 0.483 |
| AC/CC | 269 | 185 | 0.86(0.64,1.16) | 0.319 | 72 | 0.65(0.44,0.95) | 0.027 | 185 | 0.79(0.59,1.06) | 0.115 | **72** | 0.79(0.53,1.18) | 0.252 | 119 | 0.73(0.52,1.02) | 0.064 | 138 | 0.85(0.61,1.17) | 0.317 |
| *ERCC2/XPD* rs50872 | | | | | | | | | | | | | | | | | | | |
| CC | 290 | 192 | Reference |  | 77 | Reference |  | 195 | Reference |  | 74 | Reference |  | 126 | Reference |  | 143 | Reference |  |
| CT | 126 | 105 | 1.24(0.90,1.70) | 0.186 | 55 | 1.62(1.08,2.43) | 0.020 | 113 | 1.31(0.96,1.80) | 0.089 | 47 | 1.45(0.95,2.21) | 0.087 | 79 | 1.42(1.00,2.03) | 0.049 | 81 | 1.29(0.91,1.82) | 0.154 |
| TT | 14 | 15 | 1.68(0.79,3.57) | 0.180 | 6 | 1.63(0.60,4.37) | 0.336 | 16 | 1.77(0.84,3.72) | 0.134 | 5 | 1.41(0.49,4.08) | 0.522 | 10 | 1.69(0.73,3.91) | 0.223 | 11 | 1.62(0.72,3.68) | 0.247 |
| CT/TT | 140 | 120 | 1.28(0.94,1.74) | 0.113 | 61 | 1.62(1.09,2.39) | 0.017 | 129 | 1.36(1.01,1.84) | 0.046 | 52 | 1.44(0.96,2.17) | 0.082 | 89 | 1.45(1.03,2.04) | 0.032 | 92 | 1.32(0.95,1.84) | 0.104 |

* Adjusted by age and menopausal status; Ca, case; Co, control.

**Table 5. Effects of five SNPs on breast cancer risk as stratified by expression of ER, ER, and HER-2**

| **Genotype** | **Co** | **ER (-)** | | | **ER (+)** | | | **PR (-)** | | | **PR (+)** | | | **HER-2 (-)** | | | **HER-2 (+)** | | |
| --- | --- | --- | --- | --- | --- | --- | --- | --- | --- | --- | --- | --- | --- | --- | --- | --- | --- | --- | --- |
|  |  | **Ca** | **OR(95% CI)*** | ***P* value** | **Ca** | **OR(95% CI)*** | ***P* value** | **Ca** | **OR(95% CI)*** | ***P* value** | **Ca** | **OR(95% CI)*** | ***P* value** | **Ca** | **OR(95% CI)*** | ***P* value** | **Ca** | **OR(95% CI)*** | ***P* value** |
| *XPA* rs1800975 | | | | | | | | | | | | | | | | | | | |
| GG | 93 | 51 | Reference |  | 64 | Reference |  | 61 | Reference |  | 54 | Reference |  | 27 | Reference |  | 88 | Reference |  |
| GA | 231 | 86 | 0.67(0.44,1.03) | 0.067 | 149 | 0.93(0.64,1.36) | 0.718 | 109 | 0.72(0.49,1.08) | 0.113 | 126 | 0.93(0.62,1.39) | 0.720 | 46 | 0.69(0.41,1.18) | 0.177 | 189 | 0.86(0.60,1.22) | 0.399 |
| AA | 106 | 35 | 0.60(0.36,1.01) | 0.055 | 65 | 0.89(0.57,1.39) | 0.602 | 42 | 0.61(0.37,0.99) | 0.044 | 58 | 0.94(0.59,1.49) | 0.786 | 24 | 0.78(0.42,1.45) | 0.432 | 76 | 0.76(0.50,1.15) | 0.196 |
| GA/AA | 337 | 121 | 0.65(0.44,0.98) | 0.038 | 214 | 0.92(0.64,1.33) | 0.670 | 151 | 0.69(0.47,1.00) | 0.053 | 184 | 0.94(0.64,1.38) | 0.747 | 70 | 0.73(0.44,1.20) | 0.212 | 265 | 0.83(0.60,1.16) | 0.284 |
| *ERCC1* rs11615 | | | | | | | | | | | | | | | | | | | |
| CC | 261 | 98 | Reference |  | 132 | Reference |  | 119 | Reference |  | 111 | Reference |  | 47 | Reference |  | 183 | Reference |  |
| TC | 151 | 70 | 1.23(0.85,1.78) | 0.276 | 125 | 1.63(1.18,2.24) | 0.003 | 82 | 1.16(0.82,1.64) | 0.414 | 113 | 1.78(1.28,2.48) | 0.001 | 48 | 1.73(1.10,2.72) | 0.018 | 147 | 1.38(1.03,1.87) | 0.034 |
| TT | 18 | 4 | 0.58(0.19,1.76) | 0.333 | 21 | 2.28(1.17,4.44) | 0.015 | 11 | 1.34(0.61,2.94) | 0.463 | 14 | 1.81(0.87,3.77) | 0.114 | 2 | 0.66(0.15,2.95) | 0.585 | 23 | 1.80(0.94,3.44) | 0.075 |
| TC/TT | 169 | 74 | 1.16(0.81,1.66) | 0.432 | 146 | 1.68(1.24,2.29) | 0.001 | 93 | 1.17(0.84,1.64) | 0.352 | 127 | 1.76(1.28,2.43) | 0.001 | 50 | 1.61(1.03,2.51) | 0.037 | 170 | 1.41(1.06,1.88) | 0.018 |
| *XPC* rs2228000 | | | | | | | | | | | | | | | | | | | |
| CC | 228 | 84 | Reference |  | 117 | Reference |  | 96 | Reference |  | 105 | Reference |  | 41 | Reference |  | 160 | Reference |  |
| CT | 174 | 67 | 1.06(0.72,1.54) | 0.773 | 131 | 1.48(1.08,2.04) | 0.016 | 88 | 1.23(0.86,1.75) | 0.257 | 110 | 1.38(0.99,1.92) | 0.059 | 41 | 1.32(0.82,2.12) | 0.257 | 157 | 1.30(0.97,1.75) | 0.082 |
| TT | 28 | 21 | 2.27(1.20,4.26) | 0.011 | 30 | 2.15(1.22,3.78) | 0.008 | 28 | 2.62(1.45,4.73) | 0.001 | 23 | 1.83(1.00,3.34) | 0.049 | 15 | 3.09(1.50,6.36) | 0.002 | 36 | 1.93(1.12,3.31) | 0.017 |
| CT/TT | 202 | 88 | 1.21(0.85,1.72) | 0.299 | 161 | 1.58(1.16,2.14) | 0.004 | 116 | 1.41(1.01,1.96) | 0.045 | 133 | 1.44(1.05,1.98) | 0.026 | 56 | 1.55(0.99,2.42) | 0.055 | 193 | 1.39(1.04,1.84) | 0.025 |
| *XPC* rs2228001 | | | | | | | | | | | | | | | | | | | |
| AA | 161 | 72 | Reference |  | 121 | Reference |  | 94 | Reference |  | 99 | Reference |  | 43 | Reference |  | 150 | Reference |  |
| AC | 213 | 79 | 0.81(0.55,1.19) | 0.277 | 116 | 0.72(0.52,1.00) | 0.051 | 90 | 0.71(0.49,1.01) | 0.055 | 105 | 0.80(0.57,1.13) | 0.202 | 42 | 0.74(0.46,1.20) | 0.220 | 153 | 0.76(0.56,1.04) | 0.085 |
| CC | 56 | 21 | 0.80(0.45,1.42) | 0.444 | 41 | 0.97(0.60,1.55) | 0.894 | 28 | 0.81(0.48,1.37) | 0.436 | 34 | 0.99(0.60,1.62) | 0.958 | 12 | 0.77(0.38,1.58) | 0.482 | 50 | 0.94(0.60,1.47) | 0.790 |
| AC/CC | 269 | 100 | 0.81(0.56,1.16) | 0.251 | 157 | 0.77(0.57,1.05) | 0.098 | 118 | 0.73(0.52,1.02) | 0.066 | 139 | 0.84(0.61,1.16) | 0.284 | 54 | 0.75(0.48,1.18) | 0.213 | 203 | 0.80(0.60,1.07) | 0.133 |
| *ERCC2/XPD* rs50872 | | | | | | | | | | | | | | | | | | | |
| CC | 290 | 89 | Reference |  | 180 | Reference |  | 113 | Reference |  | 156 | Reference |  | 58 | Reference |  | 211 | Reference |  |
| CT | 126 | 73 | 1.86(1.28,2.71) | 0.001 | 87 | 1.10(0.79,1.53) | 0.588 | 85 | 1.71(1.20,2.43) | 0.003 | 75 | 1.09(0.77,1.55) | 0.621 | 36 | 1.43(0.89,2.28) | 0.136 | 124 | 1.33(0.98,1.81) | 0.069 |
| TT | 14 | 10 | 2.44(1.04,5.73) | 0.040 | 11 | 1.27(0.56,2.86) | 0.565 | 14 | 2.61(1.20,5.68) | 0.016 | 7 | 0.93(0.37,2.36) | 0.882 | 3 | 1.08(0.30,3.90) | 0.905 | 18 | 1.80(0.88,3.72) | 0.110 |
| CT/TT | 140 | 83 | 1.92(1.34,2.76) | 0.000 | 98 | 1.11(0.81,1.53) | 0.518 | 99 | 1.80(1.28,2.52) | 0.001 | 82 | 1.08(0.77,1.51) | 0.658 | 39 | 1.39(0.88,2.19) | 0.157 | 142 | 1.38(1.03,1.85) | 0.033 |

*Adjusted by age and menopausal status; Ca, case; Co, control.

**Table 6. Meta-analysis of the *XPC* rs2228000, rs2228001, *XPA* rs1800975 and *ERCC1* rs11615 polymorphism on breast cancer risk**

| **Variables** | **Cases/controls** | **Homozygote *vs.* wild type** | | | **Heterozygote *vs.* wild type** | | | **Dominant model** | | | **Recessive model^d^** | | |
| --- | --- | --- | --- | --- | --- | --- | --- | --- | --- | --- | --- | --- | --- |
|  |  | **OR(95% CI)** | ***P* value** | ***P_h_*^b^** | **OR(95% CI)** | ***P* value** | ***P_h_*^b^** | **OR (95% CI)** | ***P* value** | ***P_h_*^b^** | **OR(95% CI)** | ***P* value** | ***P_h_*^b^** |
| *XPC* rs2228000 | | | | | | | | | | | | | |
| Total | 3897/4877^a^ | 1.28(1.08-1.52) | 0.004 | 0.228 | 1.01(0.92,1.10) | 0.867 | 0.291 | 1.02(0.89,1.17) ^c^ | 0.766 | 0.030 | 1.25(1.06, 1.47) | 0.008 | 0.521 |
| **Ethnicities** |  |  |  |  |  |  |  |  |  |  |  |  |  |
| Caucasian | 570/676 | 0.95(0.59,1.51) | 0.811 | 0.979 | 0.86(0.68,1.08) | 0.196 | 0.983 | 0.87(0.69,1.09) | 0.219 | 0.991 | 1.01(0.64,1.59) | 0.981 | 0.990 |
| Asian | 1068/1052 | 1.73(1.30,2.31) | 0.000 | 0.384 | 1.26(1.05,1.51) | 0.015 | 0.803 | 1.37(1.15,1.64) | 0.000 | 0.475 | 1.52(1.16,1.99) | 0.002 | 0.349 |
| Other | 2259/3149 | 1.13(0.89,1.43) | 0.318 | 0.748 | 0.97(0.86,1.08) | 0.520 | 0.809 | 0.97(0.88,1.08) | 0.597 | 0.557 | 1.14(0.90,1.43) | 0.287 | 0.674 |
| **Source of control** |  |  |  |  |  |  |  |  |  |  |  |  |  |
| PB | 2364/3220 | 1.37(1.11,1.68) | 0.003 | 0.107 | 1.09(0.97,1.22) | 0.148 | 0.198 | 1.15(0.94,1.42) ^c^ | 0.171 | 0.033 | 1.29(1.06,1.57) | 0.010 | 0.266 |
| HB | 1533/1657 | 1.12(0.82,1.51) | 0.485 | 0.642 | 0.91(0.79,1.04) | 0.149 | 0.931 | 0.91(0.80,1.04) | 0.164 | 0.717 | 1.15(0.86,1.56) | 0.349 | 0.656 |
| *XPC* rs2228001 | | | | | | | | | | | | | |
| Total | 6176/6955 | 0.99(0.89,1.10) | 0.850 | 0.343 | 0.97(0.90,1.05) | 0.430 | 0.206 | 0.97(0.91,1.05) | 0.470 | 0.180 | 1.01(0.91,1.11) | 0.869 | 0.444 |
| **Ethnicities** |  |  |  |  |  |  |  |  |  |  |  |  |  |
| Caucasian | 1714/1613 | 0.85(0.70,1.05) | 0.369 | 0.369 | 0.91(0.78,1.05) | 0.194 | 0.608 | 0.89(0.78,1.03) | 0.112 | 0.462 | 0.90(0.75,1.09) | 0.279 | 0.485 |
| African | 814/753 | 0.90(0.60,1.35) | 0.649 | 0.649 | 0.94(0.77,1.16) | 0.567 | 0.308 | 0.94(0.77,1.14) | 0.512 | 0.420 | 0.93(0.63,1.37) | 0.716 | 0.513 |
| Asian | 1068/1052 | 1.14(0.87,1.49) | 0.196 | 0.196 | 1.01(0.59,1.75) ^c^ | 0.962 | 0.004 | 1.04(0.63,1.71)^c^ | 0.894 | 0.005 | 1.10(0.86,1.42) | 0.446 | 0.826 |
| Other | 2580/3537 | 1.04(0.89,1.22) | 0.298 | 0.298 | 0.99(0.88,1.10) | 0.810 | 0.792 | 1.00(0.90,1.11) | 0.998 | 0.891 | 1.06(0.92,1.21) | 0.453 | 0.130 |
| **Source of control** |  |  |  |  |  |  |  |  |  |  |  |  |  |
| PB | 4587/5222 | 0.95(0.84,1.08) | 0.441 | 0.377 | 0.95(0.87,1.04) | 0.258 | 0.081 | 0.95(0.88,1.03) ^b^ | 0.230 | 0.056 | 0.98(0.87,1.10) | 0.691 | 0.795 |
| HB | 1589/1733 | 1.10(0.89,1.35) | 0.371 | 0.336 | 1.03(0.89,1.20) | 0.700 | 0.738 | 1.05(0.91,1.21) | 0.523 | 0.971 | 1.09(0.91,1.32) | 0.346 | 0.122 |
| *XPA* rs1800975 | | | | | | | | | | | | | |
| Total | 2619/2663 | 0.92(0.65,1.31) | 0.649 | 0.003 | 1.07(0.78,1.48) | 0.663 | 0.001 | 1.03(0.74,1.42) | 0.873 | 0.000 | 0.94(0.84,1.06) | 0.303 | 0.190 |
| Ethnicities |  |  |  |  |  |  |  |  |  |  |  |  |  |
| Asian | 1407/1409 | 0.82(0.54,1.26) | 0.372 | 0.018 | 0.95(0.60,1.51) | 0.841 | 0.002 | 0.91(0.58,1.43) | 0.686 | 0.001 | 0.83(0.70,0.99) | 0.039 | 0.539 |
| Other | 1212/1254 | 1.22(0.94,1.59) | 0.129 | 0.255 | 1.30(1.01,1.66) | 0.039 | 0.821 | 1.27(1.01,1.60) | 0.046 | 0.795 | 1.05(0.89,1.23) | 0.585 | 0.239 |
| *ERCC1* rs11615 | | | | | | | | | | | | | |
| Total | 1012/1035 | 1.56(1.17,2.09) | 0.003 | 0.717 | 1.31(1.09,1.58) | 0.005 | 0.462 | 1.38(1.15,1.64) | 0.000 | 0.781 | 1.44(1.10,1.90) | 0.009 | 0.634 |

^a^rs2228000 cases/controls are not include the studies of Smith(b) and Perez-Mayoral

^b^*P_h_* value of Q-test for heterogeneity test.

^c^Random-effects model was used when a *P* value < 0.05 for heterogeneity test; otherwise, fixed-effects model was used.

^d^Available data by Tatemichi et al was used in stratified analyses by cancer type, ethnicity and source of control for the recessive model comparison.

PB: population based control studies; HB: hospital based control studies.
